# Prevalence and factors associated with food insecurity in indigenous families in the state of Alagoas (Northeast Brazil): a population-based cross-sectional study

**DOI:** 10.3389/fpubh.2025.1517746

**Published:** 2025-03-17

**Authors:** Elison Ruan da Silva Almeida, Tamara Rodrigues dos Santos, Thatiana Regina Fávaro, Ewerton Amorim dos Santos, Monica Lopes de Assunção, Haroldo da Silva Ferreira

**Affiliations:** Federal University of Alagoas, Campus A. C. Simões, Tabuleiro dos Martins, Maceió, Brazil

**Keywords:** food insecurity, malnutrition, indigenous peoples, social vulnerability, associated factors

## Abstract

**Background:**

Despite recent efforts by the government to combat Food Insecurity (FI), this issue remains a significant problem in Brazil, particularly among populations experiencing social vulnerability, such as Indigenous peoples. This study aimed to assess the prevalence and factors associated with FI in Indigenous families in the state of Alagoas.

**Method:**

It was a population-based cross-sectional survey using a probabilistic sample (*n* = 1270 families) representing the 11 ethnic groups present in the state. FI was defined according to the Brazilian Food Insecurity Scale. Factors associated with moderate and severe FI were determined through multivariable analysis using Poisson regression with robust variance adjustment (prevalence ratio – PR and 95% CI).

**Results:**

The prevalence of FI was 69.1% (39.6, 23.2, and 6.3% in mild, moderate, and severe forms, respectively). Factors associated (*p* < 0.05) with moderate and severe FI included: female-headed households; houses made of mud or wood; with ≤4 rooms; head of the household with ≤8 years of schooling; retired or unemployed household head; and total family income <2 National Minimum Wages.

**Conclusion:**

The prevalence of FI among Alagoas Indigenous peoples is considerably high and associated with poorer demographic, socioeconomic, and environmental conditions, highlighting the social inequities they face and emphasizing the need for intersectoral public policies to address this scenario.

## 1 Introduction

Food insecurity (FI) represents a severe violation of the Human Right to Adequate Food, a condition guaranteed by the Federal Constitution of Brazil and various international treaties to which the country is a signatory. FI undermines the dignity and well-being of a significant portion of Brazilian families, particularly those in a state of social vulnerability. The lack of regular and permanent access to quality food in sufficient quantities perpetuates a cycle of poverty and exclusion, limiting social and economic development, exacerbating existing inequalities, and hindering the creation of a more just and equitable society (1, 2).

FI is a multifactorial condition that affects traditional peoples and communities differently. For Indigenous peoples, the prevalence of FI is invariably higher compared to other population segments and is associated with greater social, economic, and demographic vulnerability (3, 4). Factors such as low educational attainment and changes in production methods, often imposed by territorial conflicts, further highlight the vulnerability of this population (3–5).

Indigenous peoples represent approximately 1.7 million individuals in Brazil. With an estimated population of 520,000 people, the Northeast region holds the second largest Indigenous population in the country, with approximately 25,000 of them residing specifically in the state of Alagoas (3).

The situation of FI among Brazilian Indigenous peoples has been little investigated, and there is even less research on the factors associated with this condition. The limited studies available reveal a concerning reality that reflects the social and economic inequalities faced by these peoples in Brazil (4). The Brazilian Food Insecurity Scale (EBIA), the primary tool for assessing FI in families across the country, has also been used among Indigenous peoples, but it increasingly highlights the need for adaptations to suit the diverse realities of Brazil’s Indigenous populations (6).

The population of Alagoas has historically faced significant issues such as high illiteracy rates, low income, and inadequate public infrastructure. These conditions directly impact the fulfillment of essential rights such as food, basic sanitation, and access to healthcare. Moreover, the vast cultural diversity within the same territory, as seen in Alagoas, highlights the need for carefully designed and culturally appropriate policies to address the real needs of these communities (7).

Considering the historical social vulnerability of Indigenous peoples in Brazil, and specifically in Alagoas (7), and the lack of studies focused specifically on food insecurity and understanding the factors contributing to its prevalence, this study aimed to assess the prevalence and factors associated with food insecurity in Indigenous families in the state of Alagoas. The information produced may contribute to the planning, implementation, and evaluation of actions and public policies aimed at promoting Food and Nutrition Security and ensuring the Right to Adequate Food for these peoples.

## 2 Materials and methods

### 2.1 Study design, population, and sampling

This study is a cross-sectional, population-based survey conducted as part of the Study on Nutrition, Health, and Food Security of Indigenous Peoples in the State of Alagoas (ENSSAIA). This initiative was carried out by the School of Nutrition at the Federal University of Alagoas (UFAL) in collaboration with the School of Public Health at the University of São Paulo (USP).

The ENSSAIA study evaluated a probabilistic, representative sample of Indigenous peoples in Alagoas, most of whom are distributed across the Agreste and Sertão regions of the state (Table 1).

**Table 1:** Indigenous communities in Alagoas by region, municipality, and ethnicity.

Region	Municipality	Ethnicity	Village
Agreste region	Traipu	Aconã	Aconã
	São Sebastião	Karapotó	Fazenda Terra Nova
			Plaki-ô
	Campo Grande Feira Grande	Tingui-Botó	Tingui-Botó
			Olho D’Água do Meio
Alto Sertão region	Água Branca	Kalankó	Januária
			Lajedo do Couro
			Sítio Gregório
	Inhapi	Koiunpanká	Baixa do Galo
			Roçado
			Baixa Fresca
	Pariconha	Geripankó	Ouricuri
			Figueiredo
			Moxotó
			Serra do Engenho
			Aratikun
	Pariconha	Karuazu	Tanque
			Campinhos
	Pariconha	Katokinn	Katokinn
Baixo São Francisco region	São Brás, Porto Real do Colégio	Kariri-Xokó	Kariri-Xokó
Planalto da Borborema region	Palmeira dos Índios	Xucuru-Kariri	Fazenda do Canto
			Boqueirão
			Mata da Cafuna
			Cafurna de Baixo
			Serra da Capela
			Serra do Amaro
			Coité
			Riacho Fundo
Serra dos Quilombos region	Colônia Leopoldina, Joaquim Gomes, Matriz de Camaragibe, Novo Lino	Wassú	Cocal
**Total**		**11**	**29**

Source: Adapted from the state secretariat for planning, management, and heritage (35).

Although the primary outcome of the project was food insecurity, ENSSAIA was designed as an umbrella survey aimed at addressing multiple objectives. Therefore, a conservative prevalence of 50% was adopted during the sample size planning phase. This prevalence was chosen to ensure the largest possible sample size, thereby guaranteeing robust estimates for all outcomes of interest. This approach is particularly relevant in umbrella studies, where the diversity of outcomes and heterogeneity of objectives require a broad sampling base to ensure statistical validity and generalizability of the results.

Considering a population of approximately 4,036 families (calculated by dividing the total number of Indigenous people registered in the SasiSUS in 2022 by the average household size of three individuals, according to the 2022 Census), a sampling error of 2.5%, and a 95% confidence interval, a sample of 1,113 families was determined to be necessary. It is important to note that for the outcome of “food insecurity,” the unit of analysis is the family.

In Alagoas, there are 11 Indigenous ethnic groups distributed across 29 villages. Some ethnic groups reside in a single village, while others are spread across multiple locations. To ensure representativeness and diversity in the data collected, ENSSAIA selected the necessary number of families by including one village per ethnic group. Thus, 11 villages from the 29 were randomly selected, except for the Aconã, Kariri-Xocó, Katokinn, and Wassú peoples, which each have only one village and were therefore selected with a probability of 1.

Given the defined sample size (*n* = 1,113 families; ≈ 3,339 individuals) and to provide greater balance among ethnic groups, subsamples were obtained in villages with populations exceeding 1,500 people. This was the case for the Kariri-Xocó and Wassú villages. In these cases, the entire village was not included, as was done with the other villages. Instead, sectors within the villages were randomly selected to cover approximately one-third of their respective populations. These sectors corresponded to areas registered in the SasiSUS and represented the jurisdiction of an Indigenous Health Agent (AIS).

Conversely, when the selected village represented less than one-third of the total population of a specific ethnic group, additional areas from other villages within the same ethnic group were included to meet this criterion. As a result, two villages had their number of families supplemented with residents from adjacent areas of the same ethnicity. For example, the village of Baixa Fresca was initially selected for the Koiunpanká ethnic group but was supplemented with families from the Roçado and Baixa do Galo villages. Similarly, for the Karuazu ethnic group, the Tanque village was selected and supplemented with families from the Campinhos village. Thus, although individuals from 14 localities were included in the investigation, the communities of Roçado, Baixa do Galo, and Campinhos were considered only as supplementary areas to the 11 originally selected villages.

All families residing in the selected communities were eligible for the study. Family identification was based on a registry provided by the local coordination of the Family Health Strategy, and data collection was conducted with the support and supervision of the responsible Indigenous Health Agent. The Family Health Strategy is the foundation of primary healthcare in Brazil. It utilizes multidisciplinary teams to provide continuous care to families in defined territories, with a focus on prevention and health promotion.

For this study, the sample error calculation was performed a posteriori. Considering a population of 8,575 families (3), an observed prevalence of 69.1%, a significance level of 5.0%, and a 95% confidence interval, the analyzed sample of 1,270 families corresponds to a sample error of approximately 2.4%.

### 2.2 Data collection

Data collection took place from September 2022 to December 2023 through home visits using structured forms, which were previously tested in a pilot study. Since the project aimed to include all Indigenous ethnic groups in the state, the pilot study was conducted in one of the communities (Cocal of the Wassú ethnicity), chosen for convenience (being closer to the municipality of Maceió, the capital of the state of Alagoas).

The forms were developed electronically using the Epicollect5 app, version 4.0.0, installed on tablets with Android operating systems. At the end of each interview (or at the end of the morning/afternoon shift, depending on internet availability), the completed forms were uploaded, saved in the Google account linked to each tablet. Subsequently, the spreadsheets generated by the software were transferred to a computer for the creation of the research database.

The field team consisted of 14 researchers. Considering the cultural particularities of the Indigenous peoples and to facilitate communication and participation, the team was supported by an Indigenous Health Agents, who was responsible for identifying households with registered Indigenous people in the Family Health Strategy. Additionally, an Indigenous collaborator from the village assisted the anthropometrist in transporting the equipment. Field activities were coordinated and supervised by a faculty member and a post-doctoral student with extensive experience in epidemiological surveys.

### 2.3 Variables and data collection instruments

#### 2.3.1 Dependent variable

Food insecurity (FI) was defined according to the Brazilian Food Insecurity Scale (EBIA), which comes from a scale used since the 1990s in official surveys in the USA, and has been used in Brazil for almost 20 years in official surveys, and has been adapted and validated for use in specific populations. The current version of the EBIA consists of a form with 14 closed-ended questions, with positive or negative responses regarding the family’s food experience over the past three months. Based on the number of positive responses, the scale categorizes food insecurity from concerns about potential food shortages to experiencing a full day without eating (8).

This study focused on an indigenous population with unique cultural characteristics, which required the adaptation of the EBIA. Given the lack of a validated instrument for this specific population, a version of the EBIA previously adapted for a study involving the Terena indigenous population (9) was used to better address the particularities of the studied group.

Operationally, each affirmative response on the form represents 1 point. The classification of the scale is based on the total score, ranging from 0 to 14 points in households with individuals under 18 years old, and from 0 to 8 points in households without individuals in this age group (the last 6 questions pertain to individuals under 18). Based on the score obtained, the instrument allows for classifying families as having food security (0 points) or experiencing mild, moderate, or severe food insecurity according to the following scores, respectively: 1 to 5, 6 to 9, and 10 to 14 in families with children or adolescents, and 1 to 3, 4 to 5, and 6 to 8 in those without individuals under 18 years of age. The questions included in the adapted EBIA are:

***(Questions 1, 2, 3, and 4 should be asked in all households)***

1. In the last three months, have you ever been concerned that your household’s food might run out before receiving another food basket, or before anyone in the household had money to buy food, or before you harvested produce from your own garden?
2. In the last three months, has your household’s food ever run out before the end of the month?
3. In the last three months, have you ever lacked the resources to have good food at home?
4. In the last three months, have you ever had to make do with only a few foods to eat because you did not have enough resources?
***(If the answer ‘No’ or ‘Do not Know’ is selected for all previous questions, the interview process ends. If there is at least one ‘YES,’ the next questions are applied)***
5. In the last three months, has any adult in your household, or have you personally, skipped meals (breakfast, lunch, or dinner) because there wasn’t enough food at home?
6. In the last three months, have you eaten less than you should because there was little food at home?
7. In the last three months, have you ever felt hungry and had nothing to eat at home?
8. In the last three months, have you or any adult in the household gone a whole day without eating or eaten only once a day because there was no food at home?
**[Questions 9 to 14 should be asked only in households that have residents under 18 years old (children and/or adolescents). If there are none, end the interview]**
9. In the last three months, have you been unable to provide good food to the children because the food basket had run out, there was no produce from the garden, or no money to buy food?
10. In the last three months, have the child(ren)/adolescent(s) eaten less food because there wasn’t enough food at home?
11. In the last three months, have you had to serve less food to the child(ren)/adolescent(s) because there was little food?
12. In the last three months, have the child(ren)/adolescent(s) skipped meals (breakfast, lunch, or dinner) because there wasn’t enough food at home?
13. In the last three months, have the child(ren)/adolescent(s) gone hungry and not eaten because there was no food at home?
14. In the last three months, have the child(ren)/adolescent(s) gone a whole day without eating or eaten only once a day because there was no food at home?

### 2.4 Independent variables

The independent variables related to demographic and socioeconomic conditions evaluated were: the head of household’s gender (male; female); number of people in the household (≤ 4; > 4); head of household’s education level in years of schooling (≤ 4; 5 to 8; ≥ 9); head of household’s employment status (formal employment; informal employment; retired; unemployed); total family income in number of national minimum wages (0 to 1; > 1 to 2; > 2); and family participation in a direct income transfer social program, *Bolsa Família* Program - BFP (yes; no).

Regarding environmental conditions, the variables analyzed were: type of housing (masonry; mud/wood); total number of rooms (≤ 4; > 4); garbage destination (public collection; other); source of drinking water (adequate; inadequate); and waste disposal method (adequate; inadequate).

For the source of drinking water, “adequate” conditions were considered to be: public water supply network and mineral water; while sources such as cisterns, wells, small ponds, rivers, streams, springs, trucks, or other were classified as “inadequate.” For waste disposal, “adequate” was classified as a septic tank or public sewage system, and “inadequate” was classified as a rudimentary pit, ditch, river, or other.

### 2.5 Data processing and analysis

The spreadsheets generated by the Epicollect5 software, version 4.0.0, were imported into Stata®, version 18.0, where the database was created (combine datasets) and all statistical analysis procedures were conducted. The database was initially checked for consistency, and when issues were identified, the information was reviewed to correct errors (e.g., typographical errors) or to exclude data when correction was not possible. In this sense, of a total of 1,296 families evaluated, 26 (2.0%) were excluded from the analysis due to incomplete data in the EBIA.

To enhance international comparability, the recommendations of the Food and Agriculture Organization of the United Nations (FAO) regarding food security (FS) assessment were adopted. The sum of moderate and severe food insecurity (MSFI) cases was considered as the outcome for analysis in identifying associated factors, thereby increasing the specificity of this indicator (10).

The unadjusted analysis compared the prevalence of moderate and severe food insecurity (MSFI) across different categories of independent variables (demographic, socioeconomic, and environmental). The chi-square test was employed to assess these differences. Prevalence ratios (PR) and their corresponding 95% confidence intervals (95% CI) were used as the measure of association. Both crude and adjusted prevalence ratios were calculated through Poisson regression with robust variance adjustment, which provides more accurate estimates compared to odds ratios, especially for highly prevalent conditions like food insecurity, where the odds ratio tends to overestimate the effect size.

**Figure 1:**
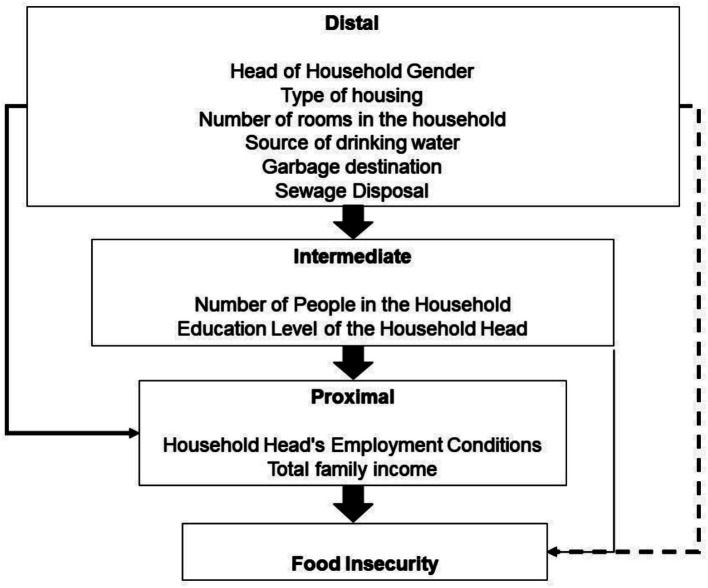
Hierarchical analysis of factors associated with moderate and severe food insecurity. Adapted from: Victora et al. (11).

Associations that showed significance at the unadjusted level of up to 20% (*p* < 0.2) were subjected to multivariable analysis, following the hierarchical conceptual model adapted from Victora et al. (11) (Figure 1).

The hierarchical analysis began with the distal level, followed by the subsequent levels according to their proximity to the outcome of the investigation. For each analysis level, variables without statistical significance were successively eliminated using backward stepwise elimination, retaining only those with *p* < 0.05. Therefore, all variables that showed p < 0.05 at their respective level, even if they exceeded the significance level in the next level, were retained in the final model.

The ENSSAIA was approved by the National Research Ethics Commission (CONEP: 29121120.0.0000.5013), and all legal requirements related to ethical procedures for conducting research involving Indigenous peoples were observed (12–14). All heads of households or eligible adults for the study, or those responsible for children or adolescents, were fully informed about the study’s objectives, risks, benefits, and other details provided in the Informed Consent Form. Participation was only allowed for those who signed the consent form.

## 3 Results

**Figure 2:**
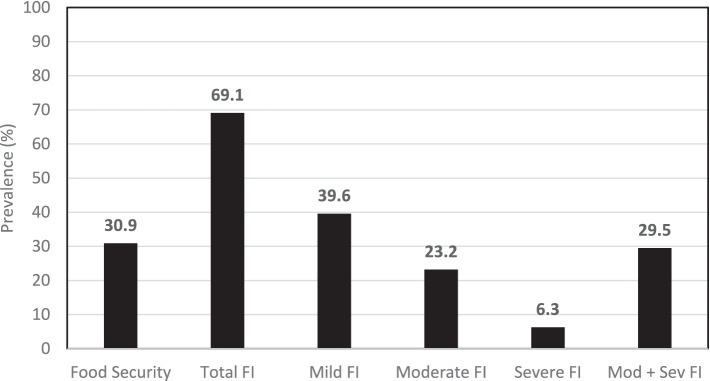
Prevalence of food security and food insecurity (FI) according to the classification of the Brazilian food insecurity scale in families from Indigenous communities in the state of Alagoas, Northeast Brazil, 2024.

The final sample consisted of 1,270 families. Most of these families were headed by men (55.5%) and consisted of up to four members (75.6%). According to the EBIA, 69.1% of the families experienced some degree of FI, with 29.5% experiencing moderate (23.2%) and severe (6.3%) FI (Figure 2).

Table 2 presents information on the population’s demographic, socioeconomic, and environmental characteristics related to the occurrence of MSFI. It was observed that, except for the source of drinking water (*p* = 0.392), all other variables were associated with MSFI at a level of significance less than 20% (*p* < 0.2), condition established for the respective variable to undergo multivariate analysis. The results indicated the social vulnerability of the families: low educational attainment of the household head (over 46% with ≤4 years of schooling), only 12.3% of heads had formal employment, and significant inadequacies in sanitation (61.8% disposed of waste inadequately). Most families were users of the *Bolsa Família* Program (57.4%).

**Table 2:** Prevalence of food insecurity (FI) according to the categories of demographic, socioeconomic, and environmental variables of indigenous communities in the state of Alagoas, Northeast Brazil, 2024.

**Variable/Category**	**Total** ***n* (%)**	**Presence of FI** ^**1**^ ***n* (%)**	**Crude PR (CI 95%)**	***p*-value**
Demographics				
Head of household gender				
Male	705 (55.51)	177 (25.11)	1	-
Female	565 (44.49)	197 (34.87)	1.39 (1.17–1.65)	< 0.001
Number of people in the household				
≤ 4	960 (75.59)	271 (28.23)	1	-
> 4	310 (24.41)	103 (33.23)	1.18 (0.97–1.41)	0.088
Socioeconomics				
Education level of the household head (in completed years of schooling)				
≥ 9	415 (32.68)	76 (18.31)	1	-
5 a 8 anos	263 (20.71)	84 (31.94)	1.74 (1.33–2.28)	< 0.001
≤ 4	592 (46.61)	214 (36.15)	1.97 (1.57–2.48)	< 0.001
Household head’s employment conditions				
Formal employment	156 (12.31)	19 (12.18)	1	-
Informal employment	472 (37.25)	126 (26.69)	2.19 (1.40–3.42)	0.001
Retired	296 (23.36)	96 (32.43)	2.66 (1.69–4.18)	< 0.001
Unemployed	343 (27.07)	132 (38.48)	3.16 (2.03–4.19)	< 0.001
Total family income (in number of national minimum wages)				
> 2	272 (21.42)	39 (14.34)	1	-
> 1 a 2	443 (34.88)	128 (28.89)	2.02 (1.46–2.79)	< 0.001
0 a 1	555 (43.70)	207 (37.30)	2.60 (1.91–3.55)	< 0.001
Family receiving assistance from the Bolsa Família program				
No	541 (42.60)	136 (25.14)	1	-
Yes	729 (57.40)	238 (32.65)	1.30 (1.08–1.55)	0.004
Environmental				
Type of housing				
Masonry	1,233 (97.09)	352 (28.55)	1	-
Mud/Wood	37 (2.91)	22 (59.46)	2.08 (1.57–2.76)	< 0.001
Number of rooms in the household				
> 4	1,133(89.21)	312 (27.54)	1	-
≤ 4	137 (10.79)	62 (45.26)	1.64 (1.34–2.01)	< 0.001
Source of drinking water				
Adequate^2^	662 (52.13)	188 (28.40)	1	-
Inadequate	608 (47.87)	186 (30.59)	1.08 (0.91–1.28)	0.392
Garbage destination				
Public collection	1,014(79.84)	287 (28.30)	1	-
Other^3^	256 (20.16)	87 (33.98)	1.20 (0.99–1.46)	0.069
Sewage disposal^4^				
Adequate	485 (38.19)	126 (25.98)	1	-
Inadequate	785 (61.81)	248 (31.59)	1.22 (1.01–1.46)	0.035

^1^FI moderate + Severe forms.

^2 General distribution network and mineral water.^

^3 Burning, vacant land, buried, or other methods.^

^4^ Adequate (Septic tank and/or sewer system), Inadequate (Primitive pit, ditch, lake, or other).

Regarding the hierarchical analysis (Table 3), the factors associated with MSFI at the distal level were: female-headed households (PR = 1.38; 95% CI: 1.16–1.63), residing in houses made of mud or wood (PR = 1.69; 95% CI: 1.25–2.28), and households with 4 or fewer rooms (PR = 1.47; 95% CI: 1.18–1.84). At the intermediate level, factors included households headed by individuals with lower educational attainment: 5 to 8 years of schooling (PR = 1.80; 95% CI: 1.38–2.34) and up to 4 years of schooling (PR = 2.02; 95% CI: 1.61–2.53). Proximal variables independently associated with MSFI included households headed by retired (PR = 1.73; 95% CI: 1.10–2.73) and unemployed individuals (PR = 1.62; 95% CI: 1.03–2.54), as well as those with a total national family income equal to or less than two National Minimum Wage - NMW (≤ 1 NMW: PR = 2.00; 95% CI: 1.44–2.76; >1 to 2 NMW: PR = 1.69; 95% CI: 1.22–2.33). At the time of data collection, the NMW in Brazil was R$ 1,320.00, approximately $275.00 USD as of July 2023.

**Table 3:** Hierarchical analysis of factors associated with the prevalence of moderate and severe food insecurity in indigenous communities in the state of Alagoas, Northeast Brazil, 2024.

Variables	Distal level		Intermediate level		Proximal level	
	PR (CI 95%)	*p*-value	PR (CI 95%)	*p*-value	PR (CI 95%)	*p*-value
Head of household gender						
Male	1		1		1	
Female	1.38 (1.16–1.63)	< 0.001	1.44 (1.23–1.71)	< 0.001	1.26 (1.04–1.51)	0.016
Type of housing						
Masonry	1		1		1	
Mud/Wood	1.69 (1.25–2.28)	0.001	1.56 (1.13–2.15)	0.007	1.53 (1.12–2.10)	0.008
Number of rooms in the residence						
> 4	1		1		1	
≤ 4	1.47 (1.18–1.84)	0.001	1.48 (1.17–1.86)	0.001	1.29 (1.02–1.63)	0.031
Garbage destination						
Public collection	1					
Others^1^	1.10 (0.88–1.36)	0.397				
Sewage disposal^2^						
Adequate	1					
Inadequate	1.14 (0.94–1.37)	0.174				
Number of people in the household						
≤ 4			1			
> 4			1.15 (0.96–1.38)	0.133		
Education level of the household head (in completed years of schooling)						
≥ 9			1		1	
5 a 8			1.80 (1.38–2.34)	< 0.001	1.55 (1.19–2.01)	0.001
≤ 4			2.02 (1.61–2.53)	< 0.001	1.78 (1.40–2.26)	< 0.001
Household head’s employment conditions						
Formal employment					1	
Informal employment					1.43 (0.91–2.22)	0.112
Retired					1.73 (1.10–2.73)	0.018
Unemployed					1.62 (1.03–2.54)	0.036
Total family income (in number of national minimum wages)^3^						
> 2					1	
> 1 a ≤ 2					1.69 (1.22–2.33)	0.001
≤ 1					2.00 (1.44–2.76)	< 0.001

^1 Burning, vacant land, buried, or other means.^

^2^ Adequate (septic tank and/or sewer system), Inadequate (rudimentary pit, ditch, lake, or other).

^3^ In Brazil, the federal minimum wage is the lowest amount a salaried worker must receive for their work performed over a month, provided they are a formal worker. Its value is set annually by a decree-law signed by the president. At the time of data collection, it was R$ 1,320.00 (approximately 275 USD at that time).

## 4 Discussion

Food insecurity is a concerning reality among Indigenous peoples in Alagoas and reflects the context of inequities in which their families are situated, underscoring the need for the development and implementation of effective cross-sectoral strategies to address the current situation. Most indigenous peoples in Alagoas are characterized by significant social vulnerability, a persistent struggle for land demarcation, and the preservation of their traditions, culture, and way of life (7). These conflicts are part of the historical experience of these peoples and are associated with structural, demographic, socioeconomic, environmental, health, and educational challenges in their communities (15, 16). Given this context, the high prevalence of FI reported is not surprising, as living conditions in households can directly or indirectly affect FI status (17).

In the observed scenario, about 40% of families showed concern or uncertainty about regular access to food, indicating a mild form of FI. Regarding more severe forms of FI (moderate and severe), nearly 30% of families faced quantitative or qualitative restrictions in their food and nutritional needs. These restrictions suggest a real possibility of these families experiencing hunger (8).

The scarcity of data in the literature on FI among Brazilian indigenous peoples, especially concerning its associated factors, contributes to the invisibility of the issues faced, perpetuating disparities and inequities compared to the rest of society. Furthermore, this lack of knowledge hampers the implementation of actions aimed at improving the quality of life for this population (4).

The comparability of this study with results reported for other indigenous communities in the country is limited due to significant cultural differences related to the concept of food security for each group, particularly regarding food access methods such as agriculture, extraction, and financial access in local markets. Additionally, comparability is also affected by differences between studies, such as participant age and data collection timing, which are strongly influenced by political processes and established public health priorities.

The EBIA is a validated scale for measuring food security in the Brazilian population, and it includes questions referring to the last three months. However, as our study focused on Indigenous peoples, we chose to use an adapted scale in which the questions referred to a 30-day period. This adaptation was applied to a small group of Teréna families from the Água Azul, Olho D’Água, and Oliveiras villages (Mato Grosso do Sul, Brazil), and we did not find other studies that used it. Therefore, in this study, we decided to maintain the three-month period to allow greater and better comparability with national and local studies that used the standard EBIA (without adaptations).

Among the available studies similar to this one, a notable study was conducted in the state of Mato Grosso do Sul involving families from the Terena ethnic group, which identified moderate to severe food insecurity (MSFI) in 53.1% of households (9). Another recently published study (4) involved indigenous families in Dourados, also in Mato Grosso do Sul, and found a total food insecurity prevalence of 64.1%, similar to the prevalence observed in the present study. It is important to highlight that there is a significant time gap between the publication of the first study and the current one, and the second study employed a different tool for defining food insecurity, known as the Short Food Security Scale.

Worldwide, households headed by women and ethnic-racial minorities are at higher risk of food insecurity (18). Although most indigenous households in Alagoas are headed by men, the situation of MSFI in female-headed households was comparatively higher (34.9% vs. 25.1%), maintaining a higher prevalence ratio at all levels in the hierarchical analysis. These results align with national and international data showing that, although most non-indigenous households are male-headed, MSFI is more prevalent in female-headed households (19–21).

In addition to gender and ethnic-racial differences, factors such as housing type, the disparity between the number of residents and rooms or living spaces, lower education of the head of the household, employment status, monthly family income, and rural versus urban housing locations have been associated with higher levels of household food insecurity in general populations. In this regard, the association between food insecurity and the factors presented in this study is consistent with what is observed in the general population, such as the association with housing quality observed in various studies (22–24).

Regarding the factors related to the environment where families live, this study points to concerning indicators of vulnerability, such as a large proportion of people without adequate access to drinking water, waste collection, and sanitation. In this context, Raupp et al. (25) confirmed the poor basic sanitation infrastructure of indigenous households in the country and reaffirmed inadequate sanitary conditions in indigenous homes in a comparative analysis between the 2000 and 2010 censuses, as well as the persistent significant racial and color inequalities in the country (24, 25).

The education level of the household head proved to be an important factor for understanding associations with food insecurity, especially when this level is below 4 or up to 8 years of schooling. In this classification, the indigenous population of Alagoas shows that over 65% of household heads fall into this category, and this variable is independently associated with FI. Similar associations have also been confirmed in the non-indigenous population with comparable numbers (26, 27). Specifically, among indigenous populations, this association was also observed among the Terena, Ñandeva, and Kaiowá in Mato Grosso do Sul and the Kaingang in the Terra de Guarita in Rio Grande do Sul (4, 28).

The association of MSFI with the absence of formal employment and lower total family income highlights the difficulties faced by this population. Bacarrin et al. (17) point out that, similar to the relationship between employment status and FI, inflation experienced by a country with high unemployment rates results in increased indicators related to FI.

In this context, and supporting the data on the income of the indigenous population in Alagoas, Brito et al. (29) found that an income below one minimum wage was associated with FI in a socially vulnerable community in São Luís, Maranhão (29). As presented in our results, nearly 60% of the families were enrolled in the PBF, a group that was significantly more exposed to food insecurity compared to families not linked to the Program. This fact underscores the effective targeting of the PBF and its importance in addressing the vulnerability situation (30).

Studies show that lower family incomes directly impact the acquisition of food in terms of quantity and quality, raising concerns about families’ access to food and compromising food security (22, 27, 29). Similarly, lower educational levels can affect financial management, ensure adequate food intake, and, more importantly, the quality of food choices for the household (26, 28). Additionally, compromised access to quality water, coupled with inadequate sanitation and waste collection services, exacerbates the conditions of families already facing a history of social vulnerability and other determinants worsening their FI (24, 25).

Due to factors such as the real increase in average worker income, the decline in the unemployment rate, and the reinstatement of effective social policies like the Bolsa Família Program, Brazil experienced a 40% reduction in the extreme poverty rate between 2022 and 2023 (31) This positively impacts purchasing power and food choices within families, establishing a favorable scenario for reducing the prevalence of food insecurity (17), However, it’s essential to recognize that socially vulnerable populations, such as Indigenous communities, Quilombolas, riverine groups, and other minority segments, still require significant attention to effectively address the persistent issue of food insecurity.

In this regard, the importance and need for studies like this one for epidemiological understanding of FI are emphasized. As the first study to assess this outcome in the indigenous population of Alagoas, it serves as a baseline for future evaluations and aids in the development and formulation of public policies that help understand and improve food security conditions in this population.

A critical issue in food insecurity scenarios is the increased exposure to conditions leading to malnutrition. Within such a context, there’s a higher likelihood of individuals experiencing malnutrition early in life, which can have lifelong consequences. This phenomenon is explained by the Developmental Origins of Health and Disease (DOHaD) theory. According to this theory, adverse exposures during the first 1,000 days of life, starting from conception, can alter a child’s growth trajectory, prioritizing immediate survival but increasing the risk of diseases later in life. Therefore, populations facing food insecurity not only confront the immediate challenges of hunger but also an elevated risk of developing chronic diseases in adulthood, the roots of which originate in the early stages of their lives (32, 33).

It is important to highlight, in this context, the role of the double burden of nutritional problems, as explained within the framework of DOHaD. Paradoxically, early-life hunger activates mechanisms that predispose individuals to the development of obesity and its comorbidities in adulthood. This paradox is further elucidated by the thrifty phenotype theory, which suggests that metabolic adaptations formed in response to early nutritional scarcity become detrimental in environments where high-calorie foods are readily available. This is particularly evident in low-income populations, where the consumption of ultra-processed foods is prevalent.

This intricate interplay of socioeconomic, dietary, and pathophysiological factors acts synergistically, undermining the population’s health and quality of life.

We recognize that the lack of access to adequate food is one of the main factors contributing to food insecurity in the studied populations. The absence of adequate resources and infrastructure prevents many families, especially in vulnerable contexts like Indigenous populations, from accessing sufficient food, directly affecting health and well-being. Awareness of the importance of healthy eating is relevant, but the central issue lies in the lack of access to nutritious food, which exacerbates health conditions and increases the risk of diseases throughout life.

In future studies, it would be important to consider not only awareness but primarily the structural and socioeconomic barriers that hinder access to healthy food. Understanding these factors will allow for the development of more effective strategies to combat food insecurity, improve health, and prevent the long-term consequences of malnutrition and its associated complications.

### 4.1 Limitations

Regarding the limitations of this study, the cross-sectional design is notable, as it does not allow for the establishment of the incidence of the problem or direct cause-and-effect relationships between variables. Additionally, the data do not permit a qualitative assessment of the specific contexts of the indigenous communities studied.

Another significant limitation is the absence of a validated measurement tool for the target population. This issue is challenging to overcome, given the great diversity of indigenous ethnicities in Brazil, each with its distinct cultures, characteristics, and different ways of accessing food.

It is essential to recognize that the specific socioeconomic, cultural, and environmental characteristics of the Indigenous population of Alagoas may influence the prevalence and factors associated with food insecurity, limiting the generalization of our findings to other contexts.

Nevertheless, this pioneering study on food insecurity among Indigenous peoples in Alagoas, provides relevant data on the magnitude and associated factors of this issue, offering valuable insights for the development of public policies and interventions targeted at this population. We believe that our findings contribute to the knowledge about the health and well-being of this population and can support actions to combat food insecurity in Indigenous communities with similar socioeconomic and cultural characteristics.

Despite the limitations presented, this study represents an important step toward understanding food insecurity among Indigenous peoples and highlights the need for specific actions to ensure the right to adequate and healthy food in this context.

## 5 Conclusion

The prevalence of FI among Indigenous people in Alagoas is considerably high especially when considering the moderate and severe levels, a condition associated with worse housing conditions, education level, access to water and sanitation, and female heads of household.

The social inequities faced by indigenous peoples, evidenced by the high prevalence of food insecurity, demand the implementation of intersectoral public policies to reverse this situation. The data presented here contribute to understanding the epidemiological profile of food insecurity and reinforce the urgency of developing strategies that consider indigenous perspectives, traditions, cultures, and ways of life. Ensuring territorial rights and strengthening sustainable family farming are essential to guarantee food access and promote the quality of life of these populations.

We reiterate the importance of studies like this one to guide, monitor, and evaluate public policies aimed at diagnosing and monitoring the conditions faced by the country’s ethnic minorities. Specifically, to address food insecurity in the short term, it is essential to strengthen and expand immediate interventions, such as income transfer programs and food distribution initiatives paired with food education programs, ensuring that these actions effectively reach Indigenous families in vulnerable situations. Furthermore, efforts to improve infrastructure, access to education, and healthcare in Indigenous communities must be prioritized as part of a broader strategy to address the structural determinants of food insecurity. These measures, combined with regular evaluations to monitor their impact, can contribute to the gradual transition of families from food insecurity to food security, promoting sustainable improvements in their quality of life (32, 34).

## Data Availability

The raw data supporting the conclusions of this article will be made available by the authors, without undue reservation.
